# A novel multiplex assay of SNP-STR markers for forensic purpose

**DOI:** 10.1371/journal.pone.0200700

**Published:** 2018-07-18

**Authors:** Tian Wei, Fei Liao, Yaowu Wang, Chao Pan, Chao Xiao, Daixin Huang

**Affiliations:** Department of Forensic Medicine, Tongji Medical College, Huazhong University of Science and Technology, Wuhan, Hubei province, China; Erasmus University Medical Center, NETHERLANDS

## Abstract

Like DIP-STR markers (deletion/insertion polymorphism-short tandem repeat combinations), SNP-STR markers (single nucleotide polymorphism-STR combinations) are also valuable in forensic DNA mixture analysis. In this study, eight SNP-STRs were selected, and a stable and sensitive multiplex polymerase chain reaction (PCR) assay was developed for amplifying these SNP-STRs and the Amelogenin gender marker according to the principle of amplification refractory mutation system (ARMS). This novel multiplex set allows detection of the minor DNA contributor in a DNA mixture of any gender and cellular origin with high resolution (beyond a DNA ratio of 1:20). In addition, SNP-STR haplotype frequencies were estimated based on a survey of 350 unrelated individuals from Chinese Han population, and the combined power of discrimination (PD) and power of exclusion (PE) of the eight SNP-STRs were calculated as 0.99999999965 and 0.9996, which were obviously higher than that of the eight STR loci: 0.9999999954 and 0.9989 respectively. The results indicated that the SNP-STR compound markers have higher application value in forensic identification compared to standard autosomal STRs, especially in the analysis of imbalanced DNA mixtures.

## Introduction

Mixed stains derived from different contributors are common biological evidence samples in forensic practice, and these complex biological samples generate mixed genotypes, presenting challenges in interpreting the results, especially for those imbalanced genomic mixtures [1, 2]. As the common forensic DNA analysis method, one of the limitations of the capillary electrophoresis (CE)-based polymerase chain reaction (PCR)-STR typing technique is that it does not work successfully if the proportion of the DNA quantities of the two contributors is more extreme than 1:10 [3]. Alternatively, Y-chromosome STRs can be used to detect the male component in these mixed samples when the DNA of the male contributor is present in a small amount [4]. However, compared with the autosomal STR analysis, the discriminatory power of Y-STR analysis is usually lower due to their paternal inheritance characteristics. So far, although a variety of strategies have been developed to separate different cell populations prior to analysis to reduce the challenges in mixture interpretation, including differential extraction [5, 6], filtration [7], fluorescence-activated cell sorting [8, 9], microchip-based separation [10–12], laser capture microdissection [13–16], micromanipulation [17–19], and microfluidic techniques [20], these methods are limited due to their complexity, low efficiency, high risk of sample cross-contamination, and/or lack of universality. Recently, massively parallel sequencing (MPS) is reported to be a promising technique for forensic mixture analysis, where all STR alleles of the minor contributors were detected in the sequence reads even for the 1% contributions [21]. In addition, MPS can also detect other types of markers, such as microhaplotype which can be highly informative for many forensic questions, including detection of DNA mixtures [22]. However, MPS is a complicated and costly technique. Therefore, there is still a need for the development of simple methods that allow complete DNA analysis of imbalanced mixtures irrespective of the gender of the DNA donors for those laboratories without NGS equipment.

In recent years, a simple solution to this problem based on CE detection platform is represented by detecting two types of compound genetic markers, deletion-insertion polymorphisms amplified with STRs (DIP-STR) [23–27] and single nucleotide polymorphisms amplified with STRs (SNP-STR) [28–30], which targets a genomic region unique to the minor DNA eliminating the masking effect of the major DNA. In comparison to SNP-STRs, although DIP-STRs are more sensitive markers (1:1,000 [24, 27] vs 1:40 [29, 30]) for the analysis of imbalanced DNA mixtures, there are still some disadvantages for forensic purpose. On one hand, DIP markers are significantly less frequent than SNPs in the human genome, and this greatly limits the selection of DIP-STR candidates. On the other hand, DIP markers are almost unavailable around the forensic commonly used STRs, such as the Combined DNA Index System (CODIS), Extended European Standard Set (ESS) and National Institute of Standards and Technology (NIST)-miniSTR, resulting in the results of DIP-STR typing are not comparable with that of routine STR typing. Based on these reasons, SNP-STRs may be more valuable compound genetic markers than DIP-STRs for the analysis of imbalanced DNA mixtures in forensic practice. The purpose of this study is to screen some valuable SNP-STR markers and develop a multiplex PCR assay, as well evaluate the application value in forensic identification, especially in the analysis of imbalanced DNA mixtures.

## Materials and methods

### DNA samples

Blood samples were collected from 350 unrelated healthy individuals of Chinese Han population in Hubei province in an anonymous way. All participants were interviewed to ensure that no individuals have common ancestry going back at least three generations. However, even so, we cannot fully exclude their distant relatedness. In addition, the peripheral blood samples of two women with singleton pregnancy (17 th and 40 th weeks respectively) and paired amniotic fluids or newborn oral swabs were also collected. Ethical approval was obtained from the medical ethics committee of Tongji Medical College of Huazhong University of Science and Technology and all individuals provided written informed consent (The informed consent for collection of the oral swabs of the female newborn was written by her mother). The control DNA 9947A (Thermo Fisher Scientific, MA, USA) was used for the multiplex assay development. Cell-free DNA of pregnant women was obtained from 2 mL of maternal plasma extracted by the QIAamp Circulating Nucleic Acid Kit (Qiagen, Hilden, Germany) according to the manufacturer’s instruction, and genomic DNA was isolated from whole blood and reference samples using the Chelex-100 method [31] and subsequently quantified with the Nanodrop 2000 spectrophotometer (Thermo Fisher Scientific, MA, USA).

### Selection of SNP-STR compound marker and primer design

Genomic databases, STRBase (https://strbase.nist.gov/) and 1000 genomes (https://www.ncbi.nlm.nih.gov/variation/tools/1000genomes/) were searched for regions containing SNP and STR polymorphisms based on the following criteria: (1) STRs are the commonly used genetic markers in forensic practice, (2) SNP and STR markers located closer than 200 bp, (3) SNP showing the minor allele frequency in Chinese Han population higher than 0.15, and (4) statistically independent SNP-STRs, such as locate on different chromosomes or on the same chromosome but the physical distance is not less than 20 Mb. According to these criteria, the first eight SNP-STRs, rs11222421-D11S4463, rs12423685-D12ATA63, rs2325399-D6S1043, rs1276598-D6S474, rs16887642-D7S820, rs9531308-D13S317, rs188010-D17S974 and rs258112-D5S2800, were selected as target compound markers in this study.

**Fig 1:**
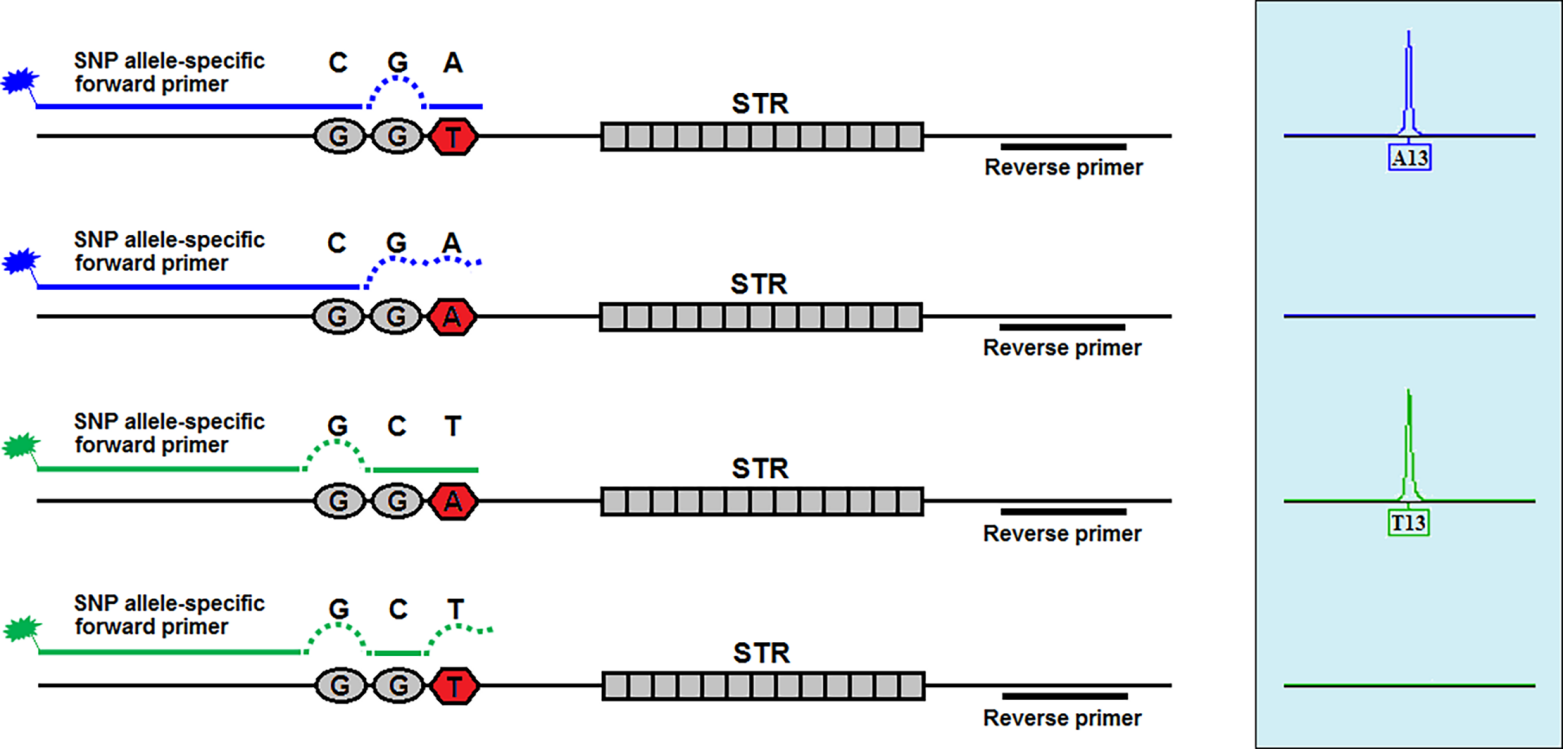
Schematic diagram of SNP-STR typing based on the principle of ARMS-PCR.

The amplification refractory mutation system (ARMS)-PCR technique [32] was used to amplify the SNP-STRs, and specificity was increased by the introduction of a deliberate mismatch at position −1, −2 or −3 of the polymorphism site. The two forward (or reverse) SNP allele-specific primers are labeled by different fluorescent dyes and the reverse (or forward) primer is located at the other flanking region of the STR which is linked to the SNP. Thus, alleles of the STR and SNP can be determined by the sizes and colors of the amplicons in one reaction respectively (Fig 1). All of primers were designed using the Primer 3 software (http://bioinfo.ut.ee/primer3/), and AutoDimer software was used to test possible primer-dimers after primer designing. All primer sequences were retested by BLAST to ensure the specificity of amplification products in the genome. In addition, we added a single G on the 5' end of the unlabeled primer within a locus-specific primer pair to promote full adenylation of PCR products amplified from that locus, and if the 5' end of the unlabeled primer was G itself, then G was not added (Table 1).

**Table 1:** Marker information, primer sequences and concentrations used in the SNP-STR multiplex assay in this study.

SNP-STR	Chromosome	SNP allele	STR repeat	Primer sequence (5′→3′) ^a^	Concentration (μM)	SNP-STR size (bp)	9947A genotype
rs11222421-D11S4463	11q25	A/T	TATC	F-A: 6-FAM-TTCTTATGAAATCTCTGTGTCTCCgA	0.090	144–180	T12, T13
				F-T: HEX-TTCTTATGAAATCTCTGTGTCTCgCT	0.065		
				R: GATAATTAAATACCATCTGAGCACTGAAGA	0.090		
rs12423685-D12ATA63	12q23.3	C/A	YAA	F: GTTGGATTTTGAGGGCCTAGGG	1.100	215–242	C13, C13
				R-C: 6-FAM-TCCCAGTTCTTTGGGAAGCaG	1.100		
				R-A: HEX-TCCCAGTTCTTTGGGAAGgTT	0.160		
rs2325399-D6S1043	6q15	C/G	AGAT	F: GCAGCTTACAGATGGCATATTGTGA	0.085	288–343	G12, C18
				R-G: 6-FAM-CATATTTTTAAGTACCCTAACAAGTAACTCAaC	0.060		
				R-C: HEX-CATATTTTTAAGTACCCTAACAAGTAACTCtTG	0.085		
rs1276598-D6S474	6q21	G/A	AGAT/GATA	F-G: 6-FAM-CATGTGTTTCTTCAGCCCCtG	0.030	375–399	A14, G18
				F-A: HEX-CATGTGTTTCTTCAGCCCgAA	0.090		
				R: GTTTGAACTTAGACTCAGCCATGC	0.090		
rs16887642-D7S820	7q21.11	G/A	GATA	F: GTCCTCATTGACAGAATTGCACC	0.230	160–184	G10, G11
				R-G: TAMRA-GTATGATAGAACACTTGTCATAGTTTAGAtC	0.170		
				R-A: ROX-GTATGATAGAACACTTGTCATAGTTTAGtAT	0.230		
rs9531308-D13S317	13q31.1	A/C	TATC	F: GACCCATCTAACGCCTATCTGT	1.600	211–239	A11, A11
				R-A: TAMRA-GTGGGGAAATTTGTACATTCATTAATATAgATT	1.400		
				R-C: ROX-GTGGGGAAATTTGTACATTCATTAATATACtTG	1.600		
rs188010-D17S974	17p13.1	T/C	CTAT	F: GACCCTGTCTCAGATAGATGGATAGG	1.800	268–296	T7, T10
				R-T: TAMRA-CCCAGAATTTAGTCTACAATTTAAAAAAGAATTtTA	0.240		
				R-C: ROX-CCCAGAATTTAGTCTACAATTTAAAAAAGAATTAaG	1.800		
rs258112-D5S2800	5q11.2	A/C	GRYW	F-A: TAMRA-ATATTACCTTCTTTATTTGATTATGTGACAaTA	2.100	339–375	C14, A23
				F-C: ROX-ATATTACCTTCTTTATTTGATTATGTGACtTTC	0.330		
				R: GTGATAGCTCAACAGGGTGACT	2.100		
Amelogenin	Xp22.1–22.3, Yp11.2	—	—	F: 6-FAM-CCCTGGGCTCTGTAAAGAATAGTG	0.018	106, 112	X, X
				R: ATCAGAGCTTAAACTGGGAAGCTG	0.018		

^a^The deliberately mismatched bases are indicated by lower case letters, and the added bases are underlined.

### PCR amplification and genotyping

PCR amplification was performed in a total reaction volume of 20 μL containing 10 μL of Platinum^®^ Multiplex Master Mix (Thermo Fisher Scientific, MA, USA), 2.4 μL GC Enhancer, 5.6 μL of the eight SNP-STRs primer mixture (Table 1) and 1 ng of DNA template. Thermal cycling was performed on GeneAmp 2720 (Thermo Fisher Scientific, MA, USA) under the following conditions: 95°C for 2 min; 30 cycles of 95°C for 30 s, 60°C for 90 s, 72°C for 35 s, and a final extension hold at 72°C for 10 min.

PCR products were electrophoresed on ABI 3130 Genetic Analyser (Thermo Fisher Scientific, MA, USA) following manufacturer’s protocols. Samples were prepared as a mixture of 0.3 μL GeneScan™ 500 LIZ^®^ size standard (Thermo Fisher Scientific, MA, USA) with 8.7 μL Hi-Di™ Formamide (Thermo Fisher Scientific, MA, USA) and 1 μL PCR products. Samples were analyzed using GeneMapper ID v3.2 software (Thermo Fisher Scientific, MA, USA) after data collection.

### Sensitivity testing

The control DNA 9947A (Thermo Fisher Scientific, MA, USA) was diluted with quantities of 1, 0.5, 0.25, 0.1, 0.05 and 0.03 ng, and each level of DNA was amplified with the multiplex system in duplicate.

### Imbalanced DNA mixtures

Based on the typing principle of the SNP-STR markers, any two samples with different informative haplotypes can be used to construct artificially DNA mixtures. Imbalanced DNA mixtures were simulated by adding increasing quantities of a major DNA to a minor DNA, and the ratios of the minor DNA to major DNA were set from 1:10, 1:20, 1:50, 1:100, 1:500 to 1:1000, keeping the level of minor contributor at 0.05 ng. Then these mixtures were genotyped using the above-mentioned multiplex amplification conditions.

### Statistical analysis

The allele frequencies and forensic parameters were evaluated using the PowerStats v1.2 software obtained from Promega [33]. Hardy–Weinberg equilibrium and pairwise linkage disequilibrium were analysed using the Arlequin v3.5 software [34]. The probability of informative genotypes (*I*) at a given SNP–STR marker was calculated according to Castella et al. [24]. The theoretical numbers of informative markers were also evaluated according to Castella et al. [24].

## Results

### Features of the selected SNP-STRs and construction of the multiplex assay

All STR loci contained in the selected eight SNP-STR compound markers are commonly used microsatellite markers: D7S820 and D13S317 are part of CODIS; D5S2800 (previous D5S2500 in NIST miniSTR 26plex and AGCU ScienTech 21-plex [35]), D6S474, D11S4463, D12ATA63 and D17S974 are part of NIST miniSTR 26plex [36]; D6S1043 is part of the commercial kits AmpFℓSTR Sinofiler™ (Thermo Fisher Scientific, MA, USA) and PowerPlex^®^ 21 (Promega, Madison, WI, USA). Six of the eight SNP-STRs locate on different chromosomes, and the other two markers, rs1276598-D6S474 and rs2325399-D6S1043, although locate on the same chromosomal arm (6q), and their physical distance and genetic distance are about 20.8 Mb and 17.73 cM (Marshfield) respectively, the pairwise linkage disequilibrium analysis showed that the two markers were genetically independent (*p* = 0.1675) in the studied Chinese Han population. For the SNPs, the minor allele frequencies of all selected loci in Chinese Southern Han population is higher than 0.2 except for the rs16887642 (0.1857) according to 1000 genomes databases (https://www.ncbi.nlm.nih.gov/variation/tools/1000genomes/).

This multiplex set was designed as a 5-dye assay, two SNP allele-specific primers for each SNP-STR marker were labeled at the 5′-end respectively with 6-FAM and HEX or TAMRA and ROX fluorescent dye for the detection by ABI 3130 Genetic Analyzer (Thermo Fisher Scientific, MA, USA). Then the eight SNP-STRs and the Amelogenin gender marker were organized by allele size ranges and assigned to each of the four dyes to achieve a single multiplex assay. After designing primers, labeling fluorescence dye and optimizing experiment conditions, a novel 9-plex fluorescent multiplex PCR system was successfully developed, and all of the eight SNP-STRs were amplified with satisfactory results (see S1 Fig). The SNP-STR markers information, primer sequences and concentrations used in our study were listed in Table 1.

### Sensitivity of the multiplex PCR assay

All of the alleles could be detected from 50 pg to 1 ng of 9947A DNA when the detection threshold was set to 50 rfu, while some alleles of a few markers could not be detected at the amount of 30 pg DNA.

### Genetic polymorphisms of the 8 SNP-STRs in Chinese Han population

The numbers of haplotypes observed in the studied population were 17, 15, 20, 10, 13, 14, 13 and 8 for rs11222421-D11S4463, rs12423685-D12ATA63, rs2325399-D6S1043, rs1276598-D6S474, rs16887642-D7S820, rs9531308-D13S317, rs188010-D17S974 and rs258112-D5S2800, respectively. These are significantly larger than the number of alleles for the corresponding STRs: 9, 10, 14, 7, 8, 8, 8 and 7, respectively. For the 8 SNPs, all of the minor allele frequency was higher than 0.2 except for the rs16887642 (0.1729) in Hubei Han population. The haplotype or allele frequencies and forensic statistical parameters for these SNP-STRs, STRs and SNPs in a Chinese Han population were shown in Table 2, and S1 and S2 Tables respectively.

**Table 2:** Haplotype frequencies and forensic statistical parameters of the 8 SNP-STRs from Hubei Han population in China (n = 350).

rs11222421-D11S4463		rs12423685-D12ATA63		rs2325399-D6S1043		rs1276598-D6S474		rs16887642-D7S820		rs9531308-D13S317		rs188010-D17S974		rs258112-D5S2800	
A9	0.0014	A15	0.0014	G10	0.0371	G15	0.0057	G8	0.0200	A7	0.0029	T6	0.0014	A17	0.2857
A12	0.0343	A16	0.0186	G11	0.1100	G16	0.0929	G9	0.0243	A8	0.2829	T7	0.0186	A18	0.2371
A13	0.0971	A17	0.1729	G12	0.1343	G17	0.1143	G9.1	0.0029	A9	0.1186	T8	0.1257	A19	0.0014
A14	0.1471	A18	0.0686	G13	0.1057	G18	0.0243	G10	0.1600	A10	0.0371	T9	0.2143	A20	0.0886
A15	0.1500	A19	0.0086	G13.2	0.0014	G19	0.0014	G11	0.3443	A11	0.0271	T10	0.0500	A21	0.0014
A16	0.0557	A20	0.0029	G14	0.1386	A12	0.0014	G12	0.2300	A12	0.0086	T11	0.0114	A23	0.0086
A17	0.0114	C11	0.0014	G15	0.0171	A14	0.3614	G13	0.0429	A13	0.0043	T12	0.0071	C14	0.3743
A18	0.0029	C12	0.3600	G19	0.0014	A15	0.3414	G14	0.0029	A14	0.0014	C8	0.0029	C18	0.0029
T9	0.0029	C13	0.0100	G20	0.0014	A16	0.0543	A8	0.1129	C9	0.0200	C9	0.0086		
T11	0.0043	C14	0.0229	C14	0.0043	A17	0.0029	A9	0.0400	C10	0.0900	C10	0.3543		
T12	0.0200	C15	0.0029	C16	0.0014			A10	0.0143	C11	0.2271	C11	0.1743		
T13	0.1400	C16	0.1886	C17	0.0571			A11	0.0043	C12	0.1343	C12	0.0300		
T14	0.1486	C17	0.1243	C17.3	0.0029			A12	0.0014	C13	0.0314	C13	0.0014		
T15	0.1143	C18	0.0157	C18	0.1657					C14	0.0143				
T16	0.0557	C19	0.0014	C18.2	0.0014										
T17	0.0129			C19	0.1614										
T18	0.0014			C20	0.0471										
				C21	0.0071										
				C22	0.0029										
				C22.3	0.0014										
*p*-value	0.6337		0.0097		0.5365		0.0936		0.6843		0.1113		0.0245		0.8550
H_obs_	0.8743		0.7600		0.9057		0.7200		0.7971		0.8543		0.7686		0.7429
H_exp_	0.8848		0.7846		0.8800		0.7286		0.7867		0.8256		0.7795		0.7151
PD	0.9731		0.9210		0.9699		0.8859		0.9245		0.9452		0.9182		0.8601
PE	0.7433		0.5270		0.8071		0.4599		0.5937		0.7033		0.5421		0.4976
*I*	0.3750		0.3181		0.3728		0.2973		0.2451		0.3747		0.3699		0.3594

*p*-value, probability of exact tests for Hardy-Weinberg disequilibrium; H_obs_, observed heterozygosity; H_exp_, expected heterozygosity; PD, power of discrimination; PE, power of exclusion; *I*, Probability of informative genotypes.

### Minor DNA detection limit in DNA mixture

**Fig 2:**
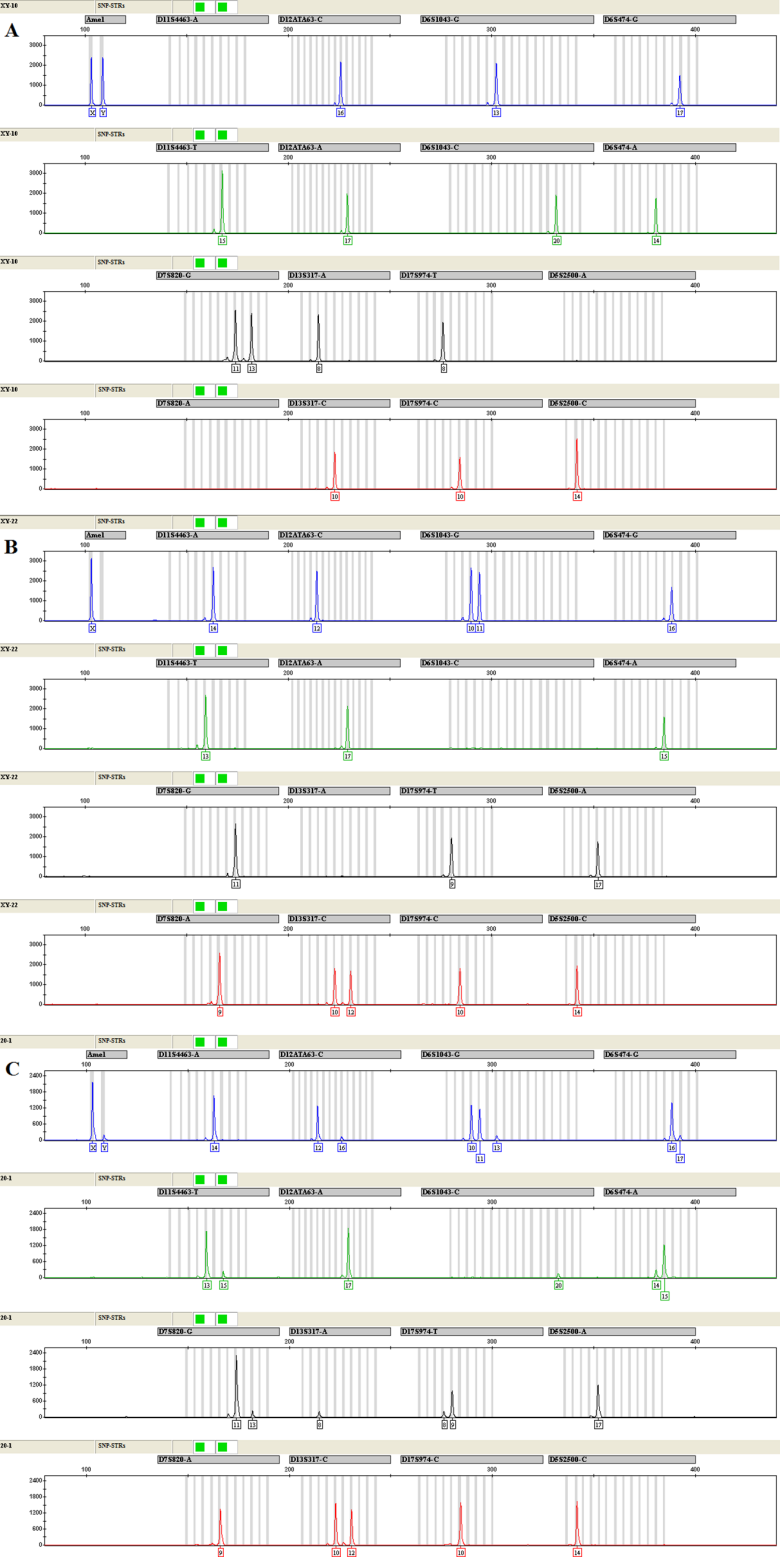
The electropherograms of the SNP-STRs multiplex assay from the sample 1 (A), sample 2 (B), and mixture mixed by samples 1 and 2 at the ratio 1:20 (C) respectively.

For the 9-plex fluorescent multiplex assay, all the markers were capable of discriminating the minor DNA up to 20-fold excess of major DNA (Fig 2). As shown in Table 3, when each marker was amplified separately in one reaction with three primers, different minor DNA detection limits for these markers were observed, ranged from 1:20 to 1:100 respectively.

**Table 3:** The minor DNA detection limit in the artificial DNA mixtures for each marker.

SNP-STRs	Detection ratios (minor: major) in different SNP subtypes of mixtures				Overall detection ratios (minor: major)
	A	G	C	T	
rs11222421-D11S4463	1:50 *			1:100	1:50
rs12423685-D12ATA63	1:50*		1:20		1:20
rs2325399-D6S1043		1:20	1:100		1:20
rs1276598-D6S474	1:100 *	1:20			1:20
rs16887642-D7S820	1:20	1:20			1:20
rs9531308-D13S317	1:50		1:100 *		1:50
rs188010-D17S974			1:100	1:50	1:50
rs258112-D5S2800	1:100 *		1:100 *		1:100

* The minor DNA could be distinguished in a mixture of 1:1000 for these SNP subtypes when they were genotyped with separate SNP allele-specific primers in two reactions and 35 PCR cycles.

### Analysis of SNP–STR markers’ performance

In the analysis of DNA mixtures, the informative genotypes denote the genotypes of minor DNA have the alleles that are absent in the genotypes of major DNA, and the probability of occurrence (*I*) is related to the allele frequency of SNP. In the present study, the *I* value for the current eight SNP–STR markers is reported in Table 2. As average, our markers show a probability of being informative of 0.3390.

**Fig 3:**
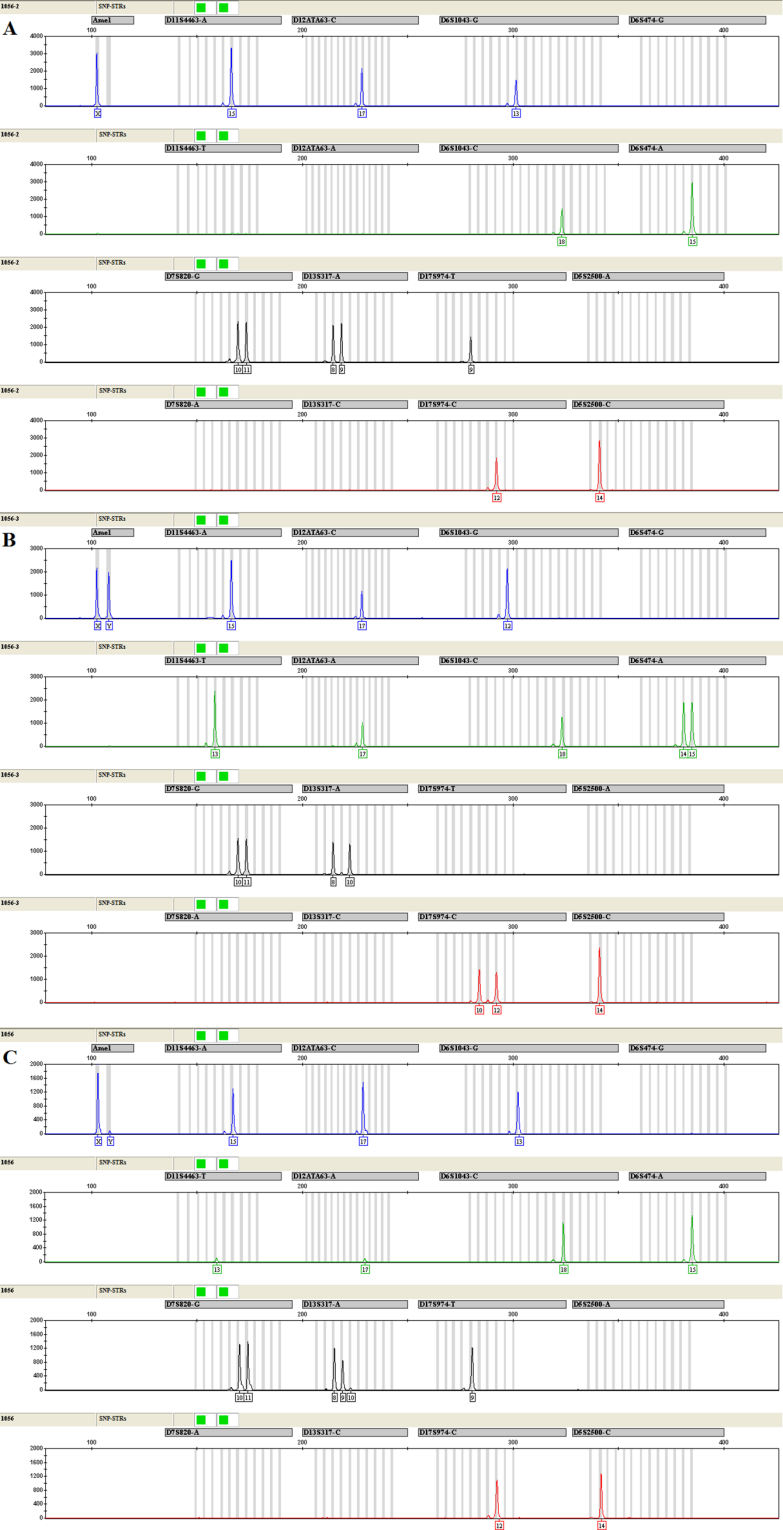
The electropherograms of the SNP-STRs multiplex assay from a woman at 17 weeks of pregnancy (A), paired amniotic fluids (B), and plasma cell-free DNA of the pregnant woman (C) respectively.

**Fig 4:**
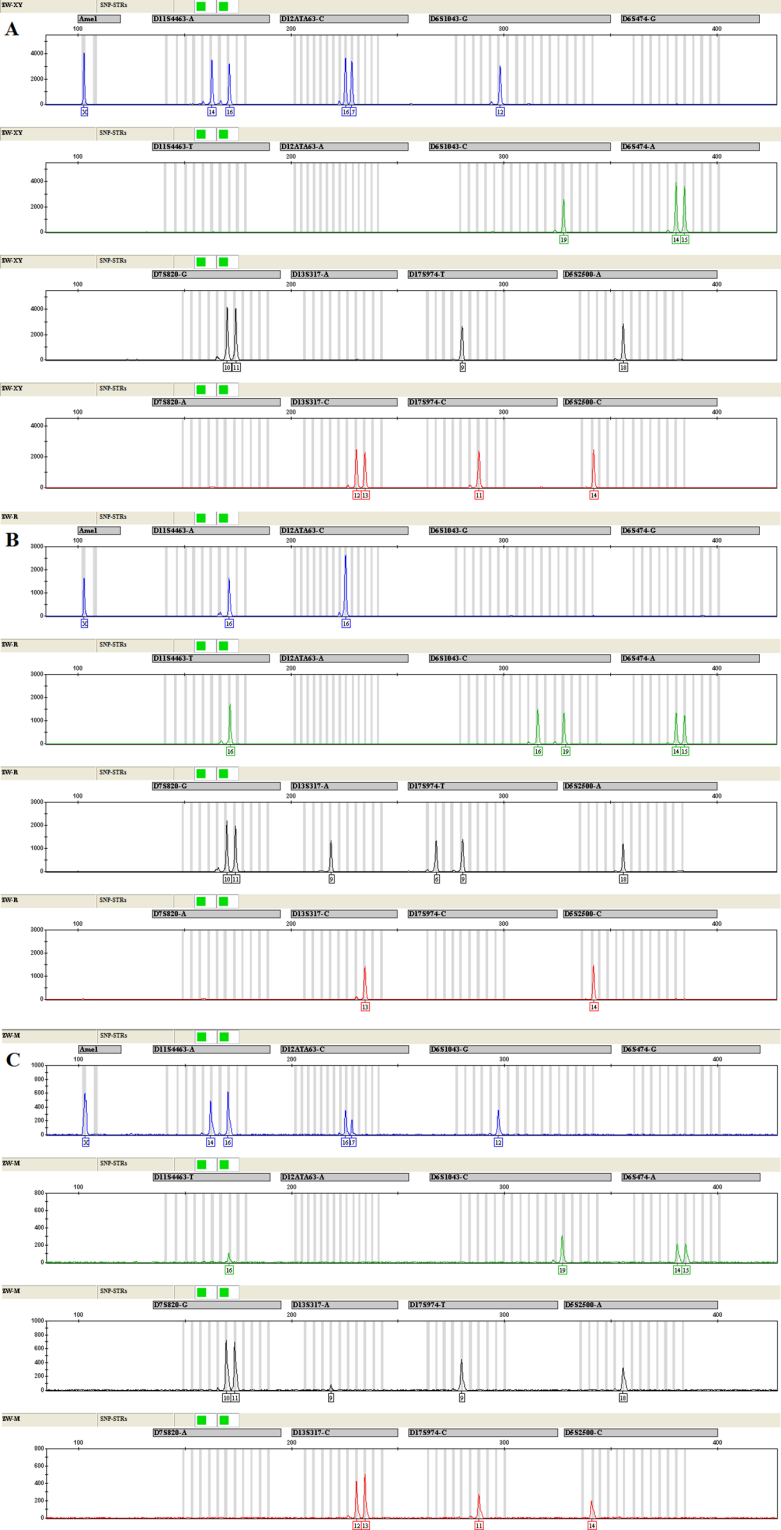
The electropherograms of the SNP-STRs multiplex assay from a woman at 40 weeks of pregnancy (A), newborn oral swab (B), and plasma cell-free DNA of the pregnant woman (C) respectively.

In addition, the typing results of pregnancy DNA microchimerism samples showed that the minor cell-free fetal DNA could be detected successfully for several informative markers (Figs 3 and 4).

## Discussion

The previous studies showed that SNP-STRs are potentially useful and valuable markers for the analysis of unbalanced genomic mixtures [28–30]. At present study, the SNP-STRs were detected according to the principle of ARMS-PCR. Through this way, after careful design and optimization of the experimental conditions, a stable and sensitive 9-plex multiplex PCR assay was developed by us, and the detection sensitivity of this assay was up to 50 pg of DNA. In forensic practice, about 0.5–1 ng DNA is routinely recommended for typing, although 50 pg DNA is enough.

In the studied Chinese Han population, the haplotypes or genotypes distributions of all SNP-STRs, STRs and SNPs markers were in accordance with Hardy-Weinberg equilibrium after Bonferroni correction (0.05/8 = 0.00625) (Table 2, and S1 and S2 Tables). In the SNP-STR compound marker, the polymorphism of SNP locus is critical for resolving DNA mixtures. For the 8 SNPs studied, there are some differences in the allele frequency distributions among several major populations in the world (see S3 Table). For example, Africans are slightly less polymorphic at these SNPs except for the rs2325399 and rs16887642 loci. In addition, it should be noted that the SNP locus rs16887642 has very low heterozygosity in Europe and the Middle East and is fixed in the relatively unadmixed Native American samples in the human genome diversity project (HGDP), while the SNPs at the other loci have reasonable heterozygosities around the world. Therefore, this will affect the application value of the assay outside of East Asia. The combined power of discrimination and power of exclusion of the eight SNP-STR compound markers were calculated as 0.99999999965 and 0.9996 in the studied population, which were obviously higher than that of the eight STR loci: 0.9999999954 and 0.9989 respectively. The results indicated that the SNP-STR compound markers have higher application value in forensic identification compared to standard autosomal STRs.

For the analysis of DNA mixtures, the SNP-STR haplotypes of minor components (0.05 ng) in the artificially imbalanced two DNA mixtures (ratio 1:20) were successfully detected using our multiplex PCR assay. However, when each SNP-STR marker was typed separately, the detection ratios of the minor DNA increased to 1:50 and 1:100 for some SNP-STRs (Table 3). Due to the introduction of deliberate mismatch at position −1, −2 or −3 of the polymorphism site, there were different amplification specificity and efficiency for different SNP allele-specific primers [37]. Therefore, for different SNP subtypes of mixtures, each SNP-STR marker may have different minor DNA detection limits. For example, for the rs11222421-D11S4463, when the samples contained A-haplotype were used as a minor component, the detection ratio was 1:50, and when the samples contained T-haplotype were used as a minor component, the detection ratio was up to 1:100 (Table 3). It is worth noting that the minor DNA could be distinguished in a mixture of 1:1000 for six different SNP subtypes in some markers when they were genotyped with separate SNP allele-specific primers in two reactions and 35 PCR cycles (Table 3), and the other 10 subtypes were not able to do it due to the interference of non-specific amplification products derived from the major DNA. In order to avoid these influence as much as possible, therefore, it is recommended that the allele-specific primers should be separately amplified when involved in the analyses of extremely imbalanced DNA mixtures.

As shown in Table 2, for these eight SNP–STR markers studied, the maximum probability of informative genotype of each locus was 0.3750, at the rs11222421-D11S4463, and the minimum was 0.2451, at the rs16887642-D7S820. As average, our markers show a probability of being informative of 0.3390. Based on the cumulative binomial distribution of these eight SNP–STR markers each one associated to a probability of being informative of 0.3390, we found that 3.64% of the mixtures have zero informative markers, 96.36% have at least one informative marker, 81.40% have at least two informative markers, and 54.56% at least three informative markers (Table 4 column 1). In Table 4 column 2, we calculated this percentage assuming the use of 30 SNP–STR markers of allele frequencies similar to the ones already developed (*I* = 0.3390). The results indicate 96.92% of DNA mixtures with at least six informative markers, 84.89% with at least eight informative markers, and 59.40% with at least 10 informative markers.

**Table 4:** Occurrence of informative markers.

Estimate using eight SNP-STR	Expected estimate using 30 SNP-STR
Percentage of DNA mixtures (≥N informative markers)	
96.36 (≥1)	96.92 (≥6)
81.40 (≥2)	92.56 (≥7)
54.56 (≥3)	84.89 (≥8)
27.04 (≥4)	73.58 (≥9)
9.39 (≥5)	59.40 (≥10)

It is well known that the plasma cell-free DNA of pregnant women is a typical imbalanced DNA mixture. In order to evaluate the application value of this multiplex assay in the analysis of imbalanced DNA mixture, two cases of pregnancy DNA microchimerism samples and paired reference samples were detected. The typing results showed that the minor cell-free fetal DNA could be detected successfully only in the Amelogenin gender marker and the SNP-STR markers with smaller amplicon sizes when there were informative haplotype differences between the mother and the fetus (Figs 3 and 4). For those SNP-STR markers with larger amplicon sizes, however, even if there were informative haplotype differences between the mother and the fetus, the cell-free fetal DNA could not be detected, which is because plasma cell-free DNA molecules are mainly short DNA fragments and the fetal DNA is shorter than maternal DNA [38, 39].

## Conclusions

In this study, a multiplex assay for detecting eight SNP-STRs and the Amelogenin gender marker was constructed. The SNP-STR haplotype of minor component (0.05 ng) in the artificially imbalanced two DNA mixture (ratio 1:20) can be detected successfully. In addition, the forensic efficiency of SNP–STRs is higher compared to standard autosomal STRs. Therefore, the SNP-STR compound markers should provide forensic scientists with a powerful tool for the analysis of DNA mixtures of any gender and cellular origin. Our future work is to develop more sets of SNP-STR markers and to derive an approach for the probabilistic evaluation of SNP-STR profiling results obtained from imbalanced DNA mixtures.

## Supporting information

S1 Fig The electropherogram of the SNP-STRs multiplex assay from the control DNA 9947A. (TIF)

S1 Table Allele frequencies and forensic statistical parameters of the 8 STRs from Hubei Han population in China (n = 350). (DOCX)

S2 Table Allele frequencies and forensic statistical parameters of the 8 SNPs from Hubei Han population in China (n = 350). (DOCX)

S3 Table Allele frequencies of the 8 SNPs in different populations. (DOCX)
