# Supplementary figures and images for: A novel multiplex assay of SNP-STR markers for forensic purpose
Source: PLoS One. 2018 Jul 18;13(7):e0200700. doi: 10.1371/journal.pone.0200700 (PMC6051632; doi:10.1371/journal.pone.0200700)

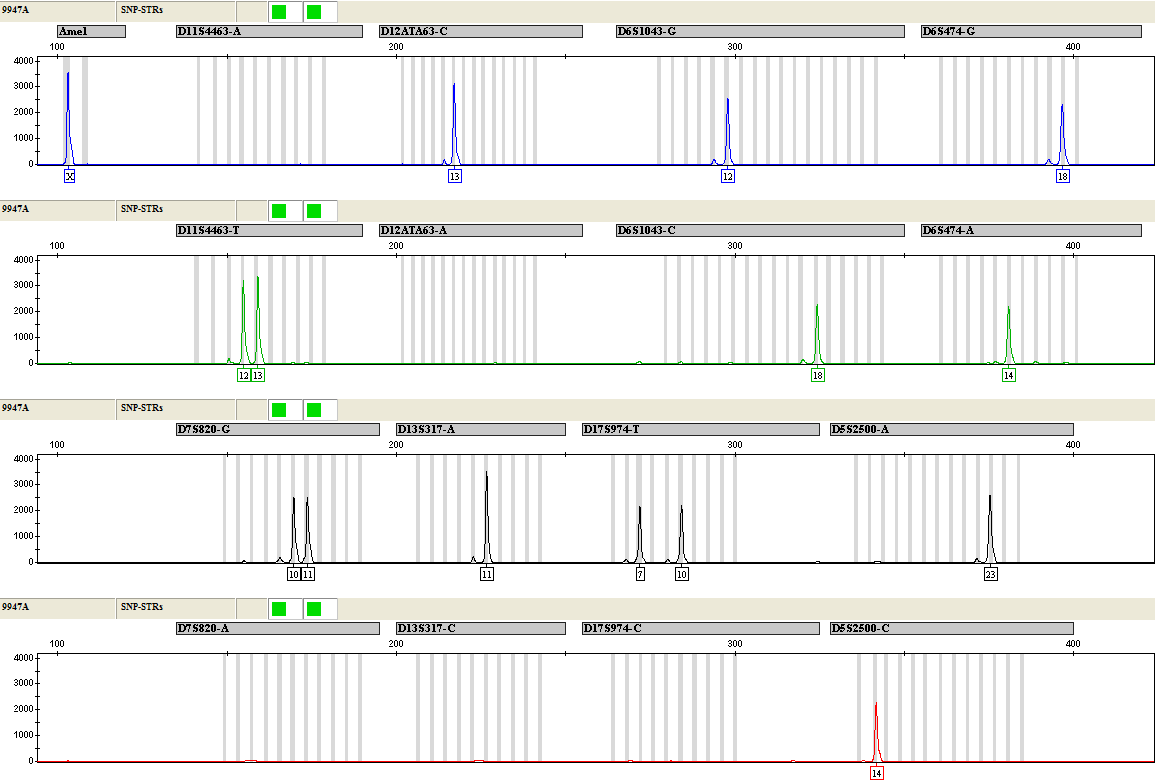

Supplement: S1 Fig — (TIF) [file pone.0200700.s001.tif]
