# Supplementary material for: A novel multiplex assay of SNP-STR markers for forensic purpose
Source: PLoS One. 2018 Jul 18;13(7):e0200700. doi: 10.1371/journal.pone.0200700 (PMC6051632; doi:10.1371/journal.pone.0200700)
Supplement: S1 Table — (DOCX) [file pone.0200700.s002.docx]

**S1 Table. Allele frequencies and forensic statistical parameters of the 8 STRs from Hubei Han population in China (n=350).**

| **D11S4463** | | **D12ATA63** | | **D6S1043** | | **D6S474** | | **D7S820** | | **D13S317** | | **D****17S974** | | **D5S2800** | |
| --- | --- | --- | --- | --- | --- | --- | --- | --- | --- | --- | --- | --- | --- | --- | --- |
| 9 | 0.0043 | 11 | 0.0014 | 10 | 0.0371 | 12 | 0.0014 | 8 | 0.1329 | 7 | 0.0029 | 6 | 0.0014 | 14 | 0.3743 |
| 11 | 0.0043 | 12 | 0.3600 | 11 | 0.1100 | 14 | 0.3614 | 9 | 0.0643 | 8 | 0.2829 | 7 | 0.0186 | 17 | 0.2857 |
| 12 | 0.0543 | 13 | 0.0100 | 12 | 0.1343 | 15 | 0.3471 | 9.1 | 0.0029 | 9 | 0.1386 | 8 | 0.1286 | 18 | 0.2400 |
| 13 | 0.2371 | 14 | 0.0229 | 13 | 0.1057 | 16 | 0.1471 | 10 | 0.1743 | 10 | 0.1271 | 9 | 0.2229 | 19 | 0.0014 |
| 14 | 0.2957 | 15 | 0.0043 | 13.2 | 0.0014 | 17 | 0.1171 | 11 | 0.3486 | 11 | 0.2543 | 10 | 0.4043 | 20 | 0.0886 |
| 15 | 0.2643 | 16 | 0.2071 | 14 | 0.1429 | 18 | 0.0243 | 12 | 0.2314 | 12 | 0.1429 | 11 | 0.1857 | 21 | 0.0014 |
| 16 | 0.1114 | 17 | 0.2971 | 15 | 0.0171 | 19 | 0.0014 | 13 | 0.0429 | 13 | 0.0357 | 12 | 0.0371 | 23 | 0.0086 |
| 17 | 0.0243 | 18 | 0.0843 | 16 | 0.0014 |  |  | 14 | 0.0029 | 14 | 0.0157 | 13 | 0.0014 |  |  |
| 18 | 0.0043 | 19 | 0.0100 | 17 | 0.0571 |  |  |  |  |  |  |  |  |  |  |
|  |  | 20 | 0.0029 | 17.3 | 0.0029 |  |  |  |  |  |  |  |  |  |  |
|  |  |  |  | 18 | 0.1657 |  |  |  |  |  |  |  |  |  |  |
|  |  |  |  | 18.2 | 0.0014 |  |  |  |  |  |  |  |  |  |  |
|  |  |  |  | 19 | 0.1629 |  |  |  |  |  |  |  |  |  |  |
|  |  |  |  | 20 | 0.0486 |  |  |  |  |  |  |  |  |  |  |
|  |  |  |  | 21 | 0.0071 |  |  |  |  |  |  |  |  |  |  |
|  |  |  |  | 22 | 0.0029 |  |  |  |  |  |  |  |  |  |  |
|  |  |  |  | 22.3 | 0.0014 |  |  |  |  |  |  |  |  |  |  |
| *p*-value | 0.7651 |  | 0.0257 |  | 0.7158 |  | 0.3306 |  | 0.6335 |  | 0.5310 |  | 0.0956 |  | 0.8071 |
| H_obs_ | 0.7600 |  | 0.7229 |  | 0.9057 |  | 0.7086 |  | 0.7800 |  | 0.8343 |  | 0.7229 |  | 0.7400 |
| H_exp_ | 0.7716 |  | 0.7324 |  | 0.8782 |  | 0.7139 |  | 0.7720 |  | 0.7992 |  | 0.7352 |  | 0.7138 |
| PD | 0.9100 |  | 0.8740 |  | 0.9692 |  | 0.8709 |  | 0.9115 |  | 0.9255 |  | 0.8902 |  | 0.8588 |
| PE | 0.5270 |  | 0.4645 |  | 0.8071 |  | 0.4416 |  | 0.5625 |  | 0.6641 |  | 0.4645 |  | 0.4928 |

*p*-value, probability of exact tests for Hardy-Weinberg disequilibrium; H_obs_, observed heterozygosity; H_exp_, expected heterozygosity; PD, power of discrimination; PE, power of exclusion.
