# Supplementary material for: A novel multiplex assay of SNP-STR markers for forensic purpose
Source: PLoS One. 2018 Jul 18;13(7):e0200700. doi: 10.1371/journal.pone.0200700 (PMC6051632; doi:10.1371/journal.pone.0200700)
Supplement: S2 Table — (DOCX) [file pone.0200700.s003.docx]

**S2 Table. Allele frequencies and forensic statistical parameters of the 8 SNPs from Hubei Han population in China (n=350).**

| **rs11222421** | | **rs12423685** | | **rs2325399** | | **rs1276598** | | **rs16887642** | | **rs9531308** | | **rs188010** | | **rs258112** | |
| --- | --- | --- | --- | --- | --- | --- | --- | --- | --- | --- | --- | --- | --- | --- | --- |
| A | 0.5000 | C | 0.7271 | C | 0.4529 | G | 0.2386 | G | 0.8271 | A | 0.4829 | T | 0.4286 | A | 0.6229 |
| T | 0.5000 | A | 0.2729 | G | 0.5471 | A | 0.7614 | A | 0.1729 | C | 0.5171 | C | 0.5714 | C | 0.3771 |
| *p*-value | 0.7495 |  | 0.0599 |  | 0.7472 |  | 0.0256 |  | 0.4516 |  | 0.3929 |  | 0.9139 |  | 0.7344 |
| H_obs_ | 0.4914 |  | 0.3571 |  | 0.5057 |  | 0.4086 |  | 0.3000 |  | 0.5257 |  | 0.4857 |  | 0.4800 |
| H_exp_ | 0.5007 |  | 0.3974 |  | 0.4963 |  | 0.3639 |  | 0.2864 |  | 0.5001 |  | 0.4905 |  | 0.4705 |
| PD | 0.6292 |  | 0.5626 |  | 0.6176 |  | 0.5215 |  | 0.4510 |  | 0.6106 |  | 0.6216 |  | 0.6042 |
| PE | 0.1801 |  | 0.0899 |  | 0.1925 |  | 0.1192 |  | 0.0635 |  | 0.2110 |  | 0.1753 |  | 0.1706 |

*p*-value, probability of exact tests for Hardy-Weinberg disequilibrium; H_obs_, observed heterozygosity; H_exp_, expected heterozygosity; PD, power of discrimination; PE, power of exclusion.
