# Supplementary material for: A novel multiplex assay of SNP-STR markers for forensic purpose
Source: PLoS One. 2018 Jul 18;13(7):e0200700. doi: 10.1371/journal.pone.0200700 (PMC6051632; doi:10.1371/journal.pone.0200700)
Supplement: S3 Table — (DOCX) [file pone.0200700.s004.docx]

**S3 Table. Allele frequencies of the 8 SNPs in different populations.**

| **Population** | **rs11222421** | | **rs12423685** | | **rs2325399** | | **rs1276598** | | **rs16887642** | | **rs9531308** | | **rs188010** | | **rs258112** | |
| --- | --- | --- | --- | --- | --- | --- | --- | --- | --- | --- | --- | --- | --- | --- | --- | --- |
|  | **A** | **T** | **C** | **A** | **C** | **G** | **G** | **A** | **G** | **A** | **A** | **C** | **T** | **C** | **A** | **C** |
| European | 0.5229 | 0.4771 | 0.6044 | 0.3956 | 0.6988 | 0.3012 | 0.4125 | 0.5875 | 0.9314 | 0.0686 | 0.6173 | 0.3827 | 0.6402 | 0.3598 | 0.7406 | 0.2594 |
| American | 0.5303 | 0.4697 | 0.6859 | 0.3141 | 0.6628 | 0.3372 | 0.4424 | 0.5576 | 0.9539 | 0.0461 | 0.6902 | 0.3098 | 0.6066 | 0.3934 | 0.8156 | 0.1844 |
| African | 0.7700 | 0.2300 | 0.9289 | 0.0711 | 0.4871 | 0.5129 | 0.1664 | 0.8336 | 0.8169 | 0.1831 | 0.8238 | 0.1762 | 0.6672 | 0.3328 | 0.8646 | 0.1354 |
| South Asian | 0.3599 | 0.6401 | 0.6534 | 0.3466 | 0.7270 | 0.2730 | 0.2955 | 0.7045 | 0.8364 | 0.1636 | 0.5297 | 0.4703 | 0.4059 | 0.5941 | 0.7321 | 0.2679 |
| East Asian | 0.5129 | 0.4871 | 0.7381 | 0.2619 | 0.5734 | 0.4266 | 0.2520 | 0.7480 | 0.8006 | 0.1994 | 0.4990 | 0.5010 | 0.4425 | 0.5575 | 0.6002 | 0.3998 |
| Hubei Han | 0.5000 | 0.5000 | 0.7271 | 0.2729 | 0.4529 | 0.5471 | 0.2386 | 0.7614 | 0.8271 | 0.1729 | 0.4829 | 0.5171 | 0.4286 | 0.5714 | 0.6229 | 0.3771 |

The data for other populations was obtained from the web site: <https://www.ncbi.nlm.nih.gov/snp/>.
